# The epidemiology and spatial distribution of *Taenia solium* taeniosis and cysticercosis in Kenya: The case of Busia County

**DOI:** 10.1371/journal.pntd.0013746

**Published:** 2025-12-05

**Authors:** Yewubdar Gulelat Zemedhun, Tadesse Egulae, Nigatu Kebede Wubie, James M. Akoko, Eric M. Fèvre, Elizabeth A. J. Cook

**Affiliations:** 1 Aklilu Lemma Institute of Health Research, Addis Ababa University, Addis Ababa, Ethiopia; 2 Department of Veterinary Sciences, College of Agriculture and Natural Resources, Dilla University, Dilla, Ethiopia; 3 International Livestock Research Institute, Nairobi, Kenya; 4 Institute of Infection, Veterinary, and Ecological Sciences, University of Liverpool, Liverpool, United Kingdom; Seoul National University College of Medicine, KOREA, REPUBLIC OF

## Abstract

**Background:**

*Taenia solium* is responsible for a substantial global disease burden in pig-raising and pork-consuming regions of the developing world. The study assessed the reported spatial pattern of *Taenia solium* taeniosis and cysticercosis (TSTC) in Kenya, with the focus on risk mapping of TSTC in Busia County.

**Methodology:**

The study employed a mixed approach, incorporating routinely collected data and open-source resources. A literature review and collection of pig population data were used to map the TSTC and pig population distribution at the national level. In Busia County, the retrospective study retrieved reports on porcine cysticercosis and epilepsy (as a proxy indicator of neurocysticercosis) from hospitals and meat inspection records. The cross-sectional study assessed risk behaviors associated with TSTC in Busia County. The TSTC reports and proportions of porcine cysticercosis and epilepsy (as a proxy indicator of neurocysticercosis) were aggregated at the county and sub-county levels and visualized using QGIS. In addition, the World Health Organization’s (WHO’s) *T. solium* mapping tool was employed for risk mapping *T. solium* in Busia County.

**Results:**

The pig population data showed a linear growth trend, with a higher population reported in Central and Western Kenya. A systematic search of the literature yielded a total of fourteen research reports, with the reported cases ranging from 1.8% to 49.9% for porcine cysticercosis, 1.6% to 31.1% for human cysticercosis, and 0.18% to 19.9% for *T. solium* taeniosis, respectively. The retrospective data showed cases of porcine cysticercosis and epilepsy (as a proxy indicator of neurocysticercosis) in all sub-counties of Busia. The WHO risk mapping tool categorized Bunyala, Teso South, Nambale, and Butula sub-counties as high-risk areas. The questionnaire survey highlighted semi-confinement as the predominant pig husbandry practice (61.1%), with 32% of pigs having access to sewage, and there was poor community awareness about TSTC in Busia County.

**Conclusion:**

The study indicated the presence of TSTC transmission in Western Kenya and identified Busia County as a high-risk area based on multiple layers of evidence. This mixed approach utilized readily available data to generate new evidence that could support sustainable One Health strategies for interrupting the *T. solium* transmission cycle.

## Author summary

The larval stage of pork tapeworm (*Taenia solium*) is the major cause of preventable epilepsy (neurocysticercosis) in humans and causes infection in pigs (porcine cysticercosis), the main intermediate host. Adult pork tapeworms result in intestinal infection (taeniosis) in humans. This study aimed to map the distribution of diseases caused by the adult and larval stages of pork tapeworm in Kenya, particularly in Busia County. The study revealed that pork tapeworm transmission is recorded in Kenya, and a relatively higher magnitude reported in the Western region. The distribution of larval pork tapeworm infection in pigs was mapped for Busia County, and sub-counties with active parasite transmission identified. Moreover, conditions favorable for maintaining adult and larval pork tapeworm transmission in Busia County were identified through interviews with the heads of pig-keeping households. The findings of this study can serve as baseline information for future control interventions targeting adult and larval pork tapeworm infection in Busia County and Kenya in a larger context.

## Introduction

*Taenia* *solium* is a zoonotic parasite responsible for a substantial global burden of disease, not only affecting human health but also resulting in a considerable economic burden for smallholder pig farmers [1,2]. Humans are the definitive host of *T. solium* and acquire the infection by ingesting undercooked pork infested with *T. solium* cysts. They can also be infected with *T. solium* eggs through fecal-oral contamination. Neurocysticercosis (NCC) occurs when larvae establish in the central nervous system [3,4]. Pigs are the main intermediate hosts, and they become infected by ingesting tapeworm eggs passed in the stool of the tapeworm carriers [3,5].

*T. solium* cysticercosis is the most common cause of preventable epilepsy in pig-raising and pork-consuming regions of the developing world [5,6]. A meta-analysis synthesized from studies conducted in Africa demonstrated that individuals infected with cysticercosis develop higher rates of epilepsy (pooled odds ratio of 3.4, 95% CI: 2.7–4.3) [7]. Similarly, a meta-analysis based on studies in Sub-Saharan Africa (SSA) [8] reported a positive association between NCC and epilepsy, with a pooled odds ratio of 2.4 (95% CI: 2.1–2.8).

The practice of keeping pigs in free-range management systems combined with an increase in pork consumption may account for the emergence of *T. solium* cysticercosis in SSA [9,10], as well as its significant contribution as a cause of epilepsy in the region [7,11,12]. This is the situation in Kenya, with an increasing preference for pork meat and a low-cost free-range husbandry system within smallholder pig-keeping communities, along with the coexistence of other determinant factors in the region [13–18].

Porcine cysticercosis-infected pigs could be an excellent indicator of local active transmission of *T. solium,* given their crucial role in the parasite life cycle and their relatively short life span and restricted range of movement [19–21]. Since *T. solium* taeniosis and cysticercosis (TSTC) occurs sporadically in clusters of infected humans and pigs [9,21,22], understanding the spatial distribution and identifying the hyperendemic foci is critical for developing cost-effective and accurately targeted control strategies [21–25].

Despite the empirical evidence on the presence of TSTC in different regions of Kenya [13–18], to the best of our knowledge, there are no summary works that have compiled the available information to show the geospatial distribution of TSTC at the country level. Hence, this study aimed to assess the reported spatial pattern of TSTC in Kenya and identify geographical areas at risk of porcine cysticercosis in Busia County.

## Methods

### Study area

The study’s scope encompasses all regions of Kenya, with a particular emphasis on Busia County. Busia County was purposefully selected for the risk mapping as the area is known for having a high pig population, based on previous epidemiological reports and favorable conditions for endemic transmission of TSTC. Busia County lies between latitudes 0^°^and 0^°^45 North and longitude 34^°^ 25 East. The county has an altitude between 1,130 and 1,375 m above sea level. It receives an annual rainfall of between 760 and 2000 millimeters and has an average yearly temperature of 22 °C (ranging from 14 °C to 30°C) [26–28]. Agriculture, fishing, and trade are the primary economic activities in the county, accounting for over 65% of its total earnings. The broad agricultural production systems in the county include crop cultivation, livestock rearing, and fisheries, characterized by a large number of small-scale and mixed crop-livestock units [29]. Busia is among the counties with a high population of pigs in Kenya [30]. The pig production system in the county is characterized as a small-scale, extensive production system [15,16,26,30–32]. The county has seven administrative sub-counties: Samia, Bunyala, Butula, Matayos, Nambale, Teso North, and Teso South.

### Study design

Different methods were used to collect data for mapping the spatial distribution of TSTC nationwide and in Busia County. At the national level, a comprehensive literature review and collection of pig population data were used to generate the TSTC distribution and pig population map. In Busia County, both retrospective and prospective approaches were employed to map the distribution of TSTC. The retrospective study retrieved reports on porcine cysticercosis and epilepsy (as a proxy for NCC) from hospitals and meat inspection records of Busia County. The prospective cross-sectional study gathered data on risk behaviors associated with TSTC transmission in Busia County. The World Health Organization’s (WHO) *T. solium* mapping tool was used to conduct a risk mapping of *T. solium* in Busia County.

### Methods of data collection

#### Collection of data on the pig population.

The pig-keeping counties of Kenya were identified by examining the livestock population data at the Directorate of Veterinary Services. The selection of pig-producing counties was further guided by the FAO Kenya pig sector report [30]. Subsequently, 20 counties were selected and visited for discussion with the veterinary officers and a review of the pig population data. The counties included Kiambu, Muranga, Kirinyaga, Nyeri, Embu, Meru, Nairobi, Nakuru, Kakamega, Trans Zoia, Kericho, Kisumu, Busia, Vihiga, Kakamega, Homabay, Migori, Kisii, Siaya, and Nairobi. In addition, we assessed the growth of pig population using data on the Kenya’s pig population from 2015 to 2021, retrieved from the FAOSTAT database [33].

#### Collection of retrospective hospital and slaughterhouse records.

After securing a research permission letter from Busia County Director of Public Health, the public health officers in all sub-counties of Busia County were contacted to collect data on routinely registered cases of *T. solium* taeniosis, human cysticercosis, and NCC between 2019 and 2021. Registered epilepsy cases were collected as a proxy indicator of NCC as recommended by WHO *T. solium* mapping tool [34], due to a lack of recorded data on NCC. The retrospective data on the registered cases of epilepsy were retrieved from a total of seven government hospitals located in 7 sub-counties of Busia, including six sub-county hospitals and one county referral hospital.

Data on the number of slaughtered pigs and condemned carcasses due to porcine cysticercosis between 2019 and 2021 were obtained from routine meat inspection records maintained by the Busia County Meat Inspection Office. Permission to access these records was granted by the County Director of Veterinary Services. The authors did not observe the meat inspection procedures at the slaughterhouse level. However, all the slaughtered pigs included in the retrospective data were inspected in accordance with the Kenya Meat Control Act [35]. The standard post-mortem inspection included palpation and incision of the tongue, visual inspection and incision of the heart, and visual inspection of all exposed muscles, especially the neck, loin, ham, and fleshy part of the diaphragm for the presence of cysts [36].

#### Collection of data for the WHO mapping protocol.

The WHO *T. solium* mapping tool [34] was employed to conduct a risk mapping of *T. solium* in Busia County. The WHO risk mapping protocol is used to identify *T. solium* hotspot areas in endemic countries by utilizing routinely collected information. This approach particularly supports *T. solium* control initiatives in developing nations where there are no adequate financial and infrastructural resources for expensive prevalence surveys [37]. The tool classifies areas with high, moderate, and low risk for active transmission of *T. solium* based on disease data and key risk factor parameters. The disease parameters include data on taeniosis and NCC/epilepsy in humans, as well as porcine cysticercosis in pigs. The risk factors considered are backyard pigs (free-roaming pigs) and open defecation/ insufficient basic sanitation [34,38].

The information used to determine these parameters can be gathered from various sources such as Ministry of Health reports, health centers, hospitals, Ministry of Livestock (or equivalent), veterinary services, slaughterhouses, local butchers, markets, street vendors, research institutes, WASH programs, ministry for Infrastructure, scientific publications, PhD and MSc theses, and more [38].

In our study, taeniosis reports from literature [39] and retrospective data on registered cases of epilepsy from county and sub-county hospitals (details of data collection explained above) were used as human disease data. Additionally, porcine cysticercosis reports by [40] and retrospective data on recorded porcine cysticercosis cases from county meat inspection records (details of data collection explained above) were included in the risk mapping. Data on free-range pigs and open defecation in each sub-county were collected through a questionnaire survey, and expert opinion from the veterinary office was also obtained. The WHO *T. solium* risk classification template used in this study is provided in S2 Data.

#### Questionnaire survey.

A semi-structured questionnaire was administered to 284 pig-keeping households to collect information on pig-keeping practices, potential risk factors, and knowledge, attitude, and practice (KAP) toward TSTC. All seven sub-counties of Busia County were used as the sampling frame. From the total of 35 wards in the county, an average of 4 to 5 wards in each sub-county (a total of 31 wards) were purposefully selected based on the number of pig-keeping households with the help of respective sub-county veterinary officers. Then, in each selected ward, the administrative chief provided a list of pig-keeping households, from which an average of 8 to 10 households were randomly chosen using a random number table. The questionnaires were written in English, programmed using ODK format, and uploaded to the ILRI ODK server to enable direct entry of answers into tablets or Android phones (S1 Data). The questionnaires were pre-tested first within the research team and then at the community level. The questionnaire was administered between October and November 2019 through personal interviews by trained data collectors with excellent skills in the local language.

#### Literature review.

Comprehensive searches were conducted to retrieve studies that investigated the magnitude, distribution, and risk factors of TSTC in Kenya and were published between the years 2000 and 2021, using the PubMed, Hinari, and Google Scholar search platforms. During the search, the boolean operators (AND, OR, and NOT) were used to combine the mesh terms with the keywords. The mesh terms and keywords used for searching include: “Porcine cysticercosis” OR “Pig tapeworm” OR “Cysticercus cellulosae” OR “*C. cellulosae”* OR “Cysticerc*” OR “*Taenia solium* cysticercosis” OR “*T. solium* cysticercosis” OR “Neurocysticercosis” OR “Taeniasis” OR “Taeniosis” OR “*Taenia solium”* AND “Kenya”. Then, the reference lists of studies included in the reviews were hand-searched for further eligible studies.

The references from the search in each database were imported directly into Mendeley’s reference manager. The titles and abstracts were then screened against the inclusion criteria, and studies conducted in Kenya, written in English, employed a cross-sectional study design, reported the magnitude of TSTC (number of infected/total number of examined), and mentioned the diagnostic methods used were retrieved in full. Then, the relevant data, including author name, publication date, country, study location, study design, sample size, diagnostic methods, and number of subjects with positive test results, were extracted from the papers into a Microsoft Excel workbook.

### Data analysis and mapping

The data collected from a questionnaire survey in Busia County were verified, coded, and analyzed using STATA 17 for descriptive statistics. For data extracted from the literature, the county administrative level was used to compile the reported TSTC cases in Kenya. For spatial mapping of TSTC, the average magnitude of reported cases were calculated and compiled by the respective county. Only the community-based cross-sectional studies were included for mapping of porcine cysticercosis, as most of the included slaughterhouse or abattoir-based studies did not specify the source region of the sampled pigs.

The proportion of epilepsy (as a proxy indicator of NCC) and porcine cysticercosis in Busia County was estimated based on the retrospective data. The data obtained from meat inspection offices and hospitals were recorded monthly. All the recorded data were aggregated at the sub-county level by summing up monthly data for each year, and then the three-year data were summed to estimate the proportion. The proportion of porcine cysticercosis cases was calculated by dividing the frequency of cases of porcine cysticercosis in the given years by the number of slaughtered and inspected pigs. For the proportion of epilepsy cases, the number of registered cases of epilepsy was divided by the total number of patients recorded in hospitals for all causes.

Then, the aggregated TSTC report and porcine cysticercosis and epilepsy proportion data were turned into county and sub-county-specific spatial layers by linking them with their respective shapefiles using QGIS. The joined shape files were then overlaid on each other to visualize the spatial distribution of TSTC reports at the country level and the proportions of porcine cysticercosis and epilepsy cases at the Busia County level. The county and sub-county shape files used in this study were obtained from the Humanitarian Data Exchange data set [41], provided by the geoBoundaries Global Administrative Database [42]. The WHO Risk Mapping Protocol [34] was used for the risk mapping of *T. solium* in Busia County.

### Ethical Considerations

Ethical approval was obtained from the Institutional Research Ethics Committee (IREC) reference (IREC2019–35) of ILRI (International Livestock Research Institute), Nairobi, Kenya (FWA00015968). Moreover, a research permit was obtained from Kenya’s National Committee for Science, Technology, and Innovations (NACOSTI), and written permission was secured from local government authorities, including the Busia County Director of Public Health and the County Director of Veterinary Services, before the start of the study. The participants of the questionnaire survey were informed about the purpose of the study. Only those who agreed to take part were recruited. In addition, written informed consent was obtained from all study participants before their involvement in the survey. Ethics also covered the collection of retrospective data from hospitals and no data was collected at the individual level.

## Results

### Pig distribution in Kenya

The pig population data retrieved from the FAOStat database showed a linear growth trend between 2015–2021 with an increase from 462,033 to over 674,764 (S1 Table). The pig population data showed Kiambu County in Central Kenya had the highest pig population, followed by Kakamega and Busia counties from the Western region, respectively.

**Fig 1:**
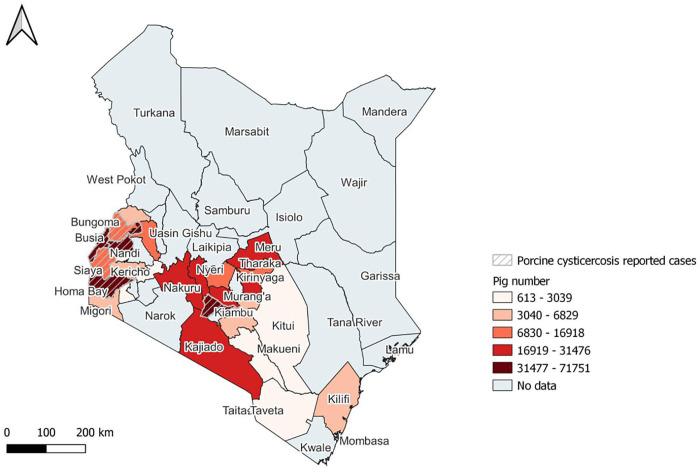
Map showing the distribution of pig population in key pig-keeping counties of Kenya and porcine cysticercosis reports.

The distribution of pig population in Kenya (from population estimates given by the County Directors of Veterinary Services (the data is presented in S3 Data)) and reports on the presence of porcine cysticercosis retrieved from published literature (Table 1) are presented in Fig 1.

**Table 1:** Summary of porcine cysticercosis case reports from 2007-2021.

Authors	Sampling year	Study location	Diagnostic method	Sample size	Magnitude of reported cases of porcine cysticercosis (95% confidence interval)
Akoko *et al.* [18]	2014	Facility based^b^	Ag-ELISA	700	8.7(6.7-11.1)
Eshitera *et al*. [16]	2010	Homa Bay County^a^	Lingual examination	392	5.6(3.6-8.4)
			Ag-ELISA	232	32.8(26.8-39.2%)
Kagira *et al.* [15]	NA	Busia County^a^	Ag-ELISA	284	3.9(1.9-6.8)
Thomas [21]	2010-2012	Busia, Kakamega, Siaya, and Bungoma counties^a^	Lingual examination	93	9.7(4.5–17.6)
Mutua *et al.* [14]	2003-2004	Teso north and Teso south sub-counties^a^ (former Teso district)	Lingual examination	505	6.5(4.0%-9.0%)
Nguhiu *et al.* [36]	2016	Thika sub-county^b^	Ag-ELISA	276	4.3(2.5-7.5)
Nguhiu *et al.* [43]	2016	Kiambu County^a^	Lingual examination	276	1.8(0.6-4.2)
Thomas *et al.* [44]	2010	Busia, Kakamega, Siaya, and Bungoma counties^b^	Lingual examination	343	5.5(3.4–8.5)
			Ag-ELISA	343	49.9(44.4–55.3)
Wardrop *et al.* [27]	2010-2012	Busia, Kakamega, Siaya, and Bungoma counties^a^	Ag-ELISA	93	17.2(10.2–26.4)
Githigia *et al.* [45]		Samia sub-county^a^ (former Funyala)	Lingual examination	107	14(8.1-22.1)
Mwabonimana *et al.* [46]	2018	Busia and Kakamega County^a^	Ag-ELISA	287	3.8(1.9-6.8)
		Busia and Kakamega County ^b^	Ag-ELISA	113	5.3(2.0-11.2)
			Meat inspection	113	1.8(0.2-6.2)

*Ag-ELISA, Antigen-based ELISA; a - community-based study; b- slaughterhouse survey; county; NA – Not available.*

The map was produced using the free and open-source QGIS software. The shapefile was obtained from Humanitarian Data Exchange Kenya Subnational Administrative Boundaries data set (https://data.humdata.org/dataset/geoboundaries-admin-boundaries-for-kenya) and provided by geoBoundaries under CC BY 4.0 license https://www.geoboundaries.org/index.html#citation [42]

### TSTC distribution in Kenya

A systematic search of the literature on the epidemiology of TSTC in Kenya resulted in eleven published articles and three grey literature sources (two PhD and one MSc thesis). Of these, eleven articles reported the porcine cysticercosis cases. Based on the included review, the reported magnitude of porcine cysticercosis ranged from 3.8% to 49.9%, 1.8% to14%, and 1.8% based on Ag-ELISA, lingual examination, and meat inspection, respectively (Table 1).

Four publications used various diagnostic methods to assess cases of human cysticercosis and *T. solium* taeniosis. The reported magnitude of human cysticercosis was 31.1% using Ab-ELISA, 2.4% upon Enzyme-linked Immunoelectro Transfer Blot assay (EITB), and 1.6-6.6% based on Ag-ELISA. The reported cases of *T. solium* taeniosis ranged from 0.18% to 6.7% based on Copro-antigen ELISA and 17.3% to 19.9% using microscopy (Table 2).

**Table 2:** Summary of *T. solium* taeniosis and cysticercosis reports in Kenya.

Authors	Sampling year	Study location	Sample size	Diagnostic method	Disease	Magnitude of reported cases (95% confidence interval)
Wardrop *et al.* [27]	2010-2012	Busia, Kakamega, Siaya, & Bungoma counties	2057	Microscopy*	HTT	0.2 (0.05-0.49)
			2057	Copro-antigen ELISA**	HTT	17.3 (15.7-19.01)
			2092	Ag-ELISA	HCC	6.6 (5.57-7.75)
Nguhiu *et al.* [47]	2016	Thika sub-county	386	Microscopy**	HTT	6.7 (4.45-9.71)
Mutua *et al.* [39]	2003-2004	Teso North and Teso South sub-counties (former *Teso district)*	6131	Microscopy**	HTT	0.18(0.09-0.32)
Downie-Ngini [48]	2006-2007	Busia County	614	Ab-ELISA	HCC	31.1 (27.5-34.94)
			614	Ag-ELISA	HCC	1.63 (0.78-2.97)
			614	EITB	HCC	2.44 (1.37- 4)

HTT – Human T. solium taeniosis; HCC – human cysticercosis; * Did not differentiate between taenia species; ** T. solium species confirmed by counting the number of uterine branches.

The spatial distribution of TSTC in Kenya was mapped based on the information obtained through the literature review (only community-based studies presented in Tables 1 and 2 were used to generate TSTC maps). Out of 47 counties in Kenya, the report on TSTC was obtained only from five counties, of which most of the TSTC studies were carried out in Western Kenya, and the rest were conducted in Central Kenya.

**Fig 2:**
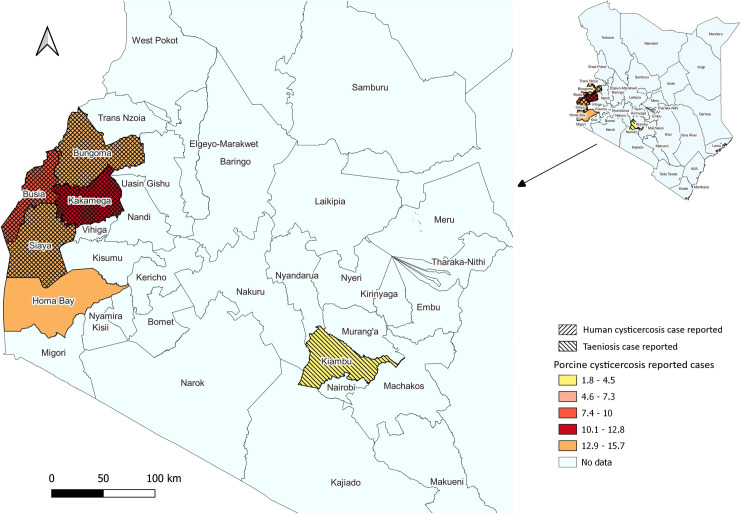
Map illustrating the spatial distribution of the reported TSTC cases in Kenya based on the literature review. The map was produced using the free and open-source QGIS software. The shapefile was obtained from Humanitarian Data Exchange Kenya Subnational Administrative Boundaries data set (https://data.humdata.org/dataset/geoboundaries-admin-boundaries-for-kenya) and provided by the geoBoundaries under CC BY 4.0 license https://www.geoboundaries.org/index.html#citation [42].

The spatial mapping (Fig 2) revealed the presence of *T. solium* taeniosis, human cysticercosis, and porcine cysticercosis in the Western region of Kenya, with Kakamega county having the highest average report of porcine cysticercosis, followed by Busia, Siaya, and Bungoma counties, respectively. The highest average porcine cysticercosis report was obtained from Homa Bay, but no report was found on either *T. solium* taeniosis or human cysticercosis from the county. The magnitude of the reported porcine cysticercosis cases is shown by a graduated color legend (Fig 2). Kiambu County had the highest pig population (Fig 1); however, the case of porcine cysticercosis was reported to be the lowest (Fig 2) with modest *T. solium* taeniosis burden (Table 2).

### The distribution of TSTC in Busia County

The retrospective meat inspection data revealed porcine cysticercosis cases in all Busia sub-counties. There was no registered data on the hospitals’ case records directly addressing the cases of *T. solium* taeniosis or NCC. Instead, cases of epilepsy were employed as a proxy indicator of NCC as described in the methodology section. The retrospective hospital data showed the recorded cases of epilepsy in all sub-counties. The highest proportion of both epilepsy (1.2%) and porcine cysticercosis cases was recorded in the Bunyala sub-county (16.6%) (Table 3).

**Table 3:** The proportion of epilepsy and porcine cysticercosis cases in Busia County based on three years of retrospective data.

Sub-county	Number of epilepsy cases (2019–2021)	Number of people visited the hospital (2019–2021)	Proportion of epilepsy cases (95% confidence interval)	No of PCC cases (2019–2021)	No of pigs slaughtered and inspected (2019–2021)	Proportion of PCC cases (95% confidence interval)
Bunyala	755	64379	1.2% (1.09 - 1.26)	368	2221	16.6% (15.05 - 18.18)
Butula	704	64886	1.1% (1.01 - 1.17)	424	2881	14.7% (13.44 - 16.06)
Teso South	106	69435	0.2% (0.13 - 0.18)	165	1018	16.2% (14 - 18.62)
Teso North	518	318103	0.2% (0.15 - 0.18)	239	2874	8.3% (7.33 - 9.39)
Matayos	1132	537706	0.2% (0.19 - 0.22)	505	4496	11.2% (10.3 - 12.19)
Nambale	402	131760	0.3% (0.28 - 0.34)	213	1656	12.9% (11.29 - 14.57)
Samia	188	40146	0.5% (0.4 - 0.54)	638	6797	9.4% (8.7 - 10.1)
Total	3805	1,226,415	0.3%	2552	21943	11.6%

**Fig 3:**
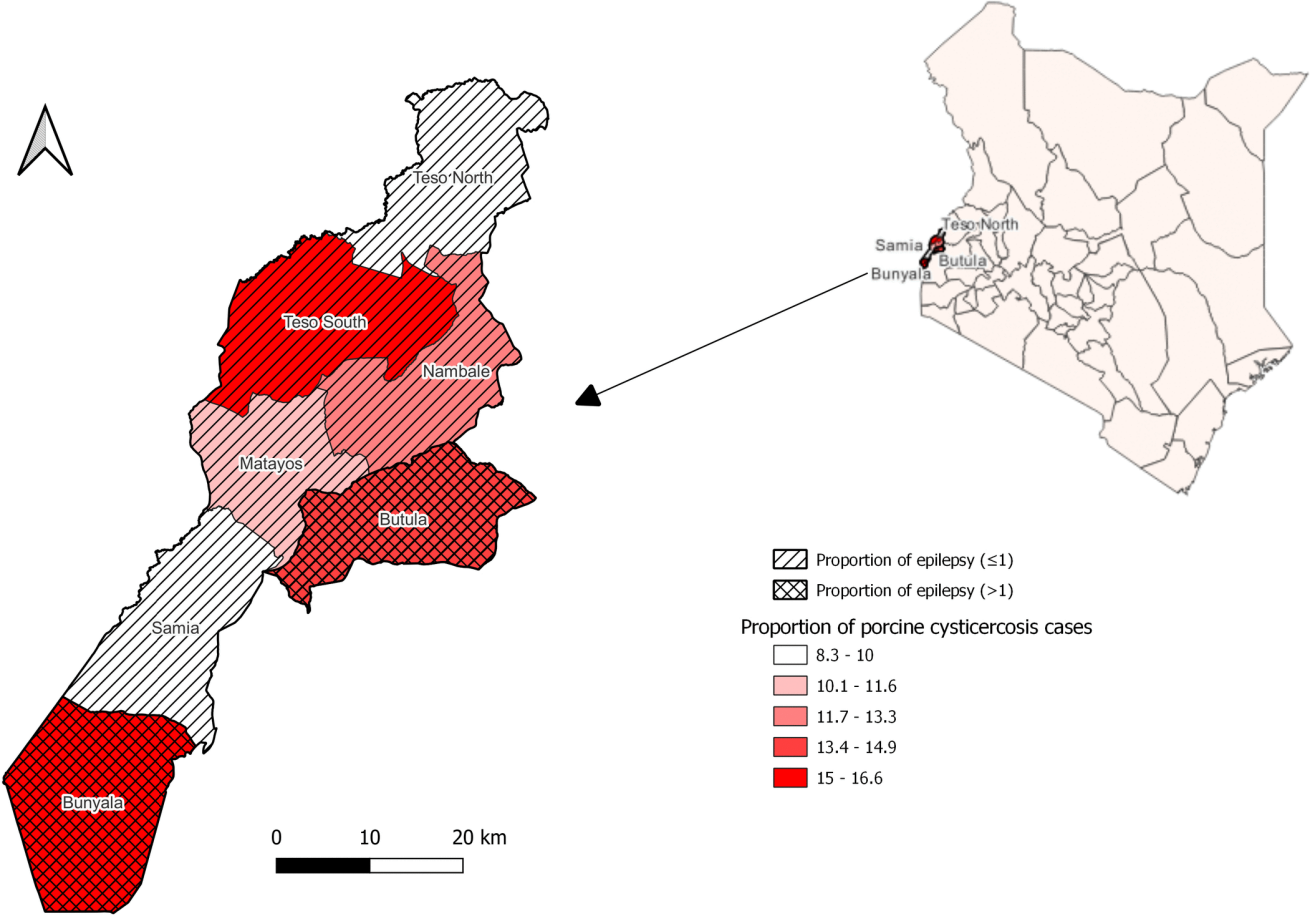
The map showing the distribution of porcine cysticercosis and epilepsy in Busia sub-counties from retrospective hospital and meat inspection records. The map was produced using the free and open-source QGIS software. The shapefile was obtained from Humanitarian Data Exchange Kenya Subnational Administrative Boundaries data set (https://data.humdata.org/dataset/geoboundaries-admin-boundaries-for-kenya) and provided by the geoBoundaries under CC BY 4.0 license https://www.geoboundaries.org/index.html#citation [42].

Fig 3 shows that Bunyala and Butula sub-counties had a relatively higher proportion of both porcine cysticercosis and epilepsy. In contrast, the Teso-North and Samia sub-counties recorded the lowest proportion of both cases.

### Questionnaire survey

Data were collected from 284 pig-keeping households across the seven sub-counties of Busia County. The majority of respondents were male (62.3%). Over half of the study participants (53.9%) had completed primary education and were between 25 and 44(55%), with 32% aged 35–44 and 23.2% aged 25–34. All respondents identified as followers of the Christian religion. The detailed sociodemographic characteristics are provided in the supplementary material (S2 Table).

The survey revealed that the semi-confined production system was the predominant pig husbandry system (61.1%) in Busia County, followed by free-range (21.6%) and the confined production method (17.3%) (Table 4). At the sub-county level, the highest proportion of semi-confined pig production systems was recorded in the Samia sub-county (77.5%). The majority of the respondents from the Bunyala sub-county practiced free-range pig production (48.6%). In contrast, the respondents from the Matayos sub-county were the lowest for keeping free-range pigs (7%) and the highest for practicing confined pig production methods (50%).

**Table 4:** Pig keeping and risk practices for taeniosis and human cysticercosis in Busia County.

Pig keeping practice and associated risk factor for TSTC	Category	Sub-county (%)							County level (%)
		Bunyala (n = 35)	Butula (n = 44)	Nambale (n = 41)	Teso South (n = 42)	Teso North (n = 41)	Samia (n = 41)	Matayos (n = 40)	
Production method	Confined	17.1	13.6	20	2.3	9.8	10	50	17.3
	Semi-confined	34.3	63.6	57.5	74.4	73.2	77.5	42.5	61.1
	Free-range	48.6	22.7	22.5	23.3	17.1	12.5	7.5	21.6
Pig access to sewage	Yes	20	11.4	22.5	48.8	53.7	47.5	17.5	31.8
	No	31.4	47.7	40	44.2	41.5	47.5	70	46.3
	Unknown	48.6	40.9	37.5	7	4.9	5	12.5	21.9
Dewormed pigs	Yes	60	67.4	72.5	86	75.6	75	75	73.4
	No	40	32.6	27.5	14	24.4	25	25	26.6
Pigs/pork market outlet	Butcher	57.1	70.5	64.1	73.8	73.2	75.6	59	68
	Local market	22.9	15.9	15.4	7.1	2.4	9.8	12.8	12.1
	Butcher & local market	14.3	9.1	12.8	19.1	14.6	2.4	17.9	12.8
	Other	5.7	4.5	7.7	0	9.8	12.2	10.3	7.1
Consume pork	Yes	71.4	81.4	62.5	76.7	75.6	77.5	82.5	75.5
	No	28.6	18.6	37.5	23.3	24.4	22.5	17.5	24.5
Main pork source	Butcher	97.1	95.3	80	80	97.6	90	97.3	90.9
	Other	2.9	4.7	20	20	2.4	10	2.7	9.1
Cooking preference	Frying	40	72.2	56	66.7	71	77.4	66.7	65.7
	Boiling and frying	56	19.4	40	27.3	29	19.4	33.3	31
	Boiling	0	2.8	0	6.1	0	0	0	1.4
	Grilling	0	2.9	0	0	0	0	0	0.5
	Other	4	2.8	4	0	0	3.2	0	1.4
Home slaughter	Yes	22.9	25.6	15	7	9.8	32.5	7.5	17
	No	77.1	74.4	85	93	90.2	67.5	92.5	83
Presence of latrine	Yes	77.1	95.3	95	95.3	97.6	95	95	93.3
	No	22.9	4.7	5	4.7	2.4	5	5	6.7
Type of latrine	Completely closed	7.4	29.3	23.7	39	52.5	21.1	18.4	28.5
	Partially closed	40.7	31.7	23.7	7.3	10	0	5.3	16
	Open pit	51.9	39	52.6	53.7	37.5	78.9	76.3	55.5
Water access after using toilet	Yes	23.5	60.5	67.5	53.5	70.7	75.0	47.5	57.7
	No	76.5	39.5	32.5	46.5	29.3	25	52.5	42.3
Washing hand before each meal	Yes	67.9	75.0	97.2	88.2	88.2	91.7	81.5	85
	No	32.1	25	2.8	11.8	11.8	8.3	18.5	15
Wash/peel vegetables and fruits before eating raw	Yes	91.4	83.7	100	85.7	95.1	92.5	85	90.4
	No	8.6	16.3	0	14.3	4.9	7.5	15	9.6
Dewormed against the GIT parasite	Yes	60	67.4	72.5	86	75.6	75	75	73.4
	No	40	32.6	27.5	14	24.4	25	25	26.6
**Color code** based on WHO *T. solium* risk mapping of Busia									

Nearly one-third (31.8%) of respondents in Busia County reported that their pig had access to sewage. On the other hand, nearly half of the respondents from Teso South (48.8%) reported the same. Most respondents in Busia County (73.4%) had dewormed their pigs at least once a year. At the sub-county level, the highest proportion of respondents who dewormed their pigs was recorded in the Teso South sub-county (86%).

The respondents were also asked about their pork consumption and 75.5% reported that they eat pork. From pork-consuming respondents, frying (65.7%) was identified as a popular cooking method, followed by boiling and frying (31%). Grilling was reported as the least preferred cooking method practiced by 0.5% of the respondents. Most households responded that the local butcher shops (94%) were their primary source of pork, while the rest reported that they access pork from different sources (local brew shop or village, home, etc.). This is also the same at the sub-county level, with 80-97.1% of respondents accessing pork from local butcher shops. Home slaughter was practiced by 17% of respondents at least once, while 83% never slaughtered pigs in their homestead. The practice of home slaughter was found to be the highest (32.5%) in the Butula sub-county.

Ninety-three percent of respondents reported having latrines in their household, of which the majority had open pit latrines (55.5%), followed by completely closed (28.5%) and partially closed privy (16%). Compared to other sub-counties, the Butula sub-county had the highest percentage of respondents (22.9%) who did not have access to a latrine in their household. More than half (55.5%) of respondents have access to water after using the toilet. Hand washing was done by 85% of participants before meals, and 90.4% of respondents reported washing or peeling vegetables and fruits before eating them raw. The majority (73.4%) of respondents reported self-deworming at least once a year.

The majority (60%) of respondents in the Matayos sub-county heard about pork tapeworm, and 54.2% addressed the transmission route. In contrast, 42.9% of Bunyala sub-county respondents heard about porcine cysticercosis, but most of them (80%) could not explain its means of transmission (Table 5).

**Table 5:** Knowledge attitude and practice of participants on the transmission of TSTC in Busia County.

KAP of participant on TSTC transmission	Sub-county (%)							
	Bunyala	Butula	Nambale	Teso South	Teso North	Samia	Matayos	Busia county
Knew/heard of PCC	42.9	56.8	53.7	46.5	39	47.5	60	49.6%
Associated PCC with pigs scavenging on human feces/ contaminated pasture	20	52	65	45	43.8	36.8	54.2	46.8%
Knew/heard of HTT	57.1	27.9	60	62.8	65.9	45	37.5	50.7%
Associated HTT with the consumption of undercooked pork	5	25	12.5	40.7	55.6	16.7	33.3	28.7%
Associated HTT, HCC and PCC	5.7	0	2.5	7	19.5	15	7.5	8.2%
**Color code** based on WHO *T. solium* risk mapping of Busia	High				High	Moderate	High	

HTT – Human *T. solium* taeniosis; HCC – human cysticercosis; PCC – Porcine cysticercosis

Half of the respondents in Busia County (50.7%) heard about *T. solium* taeniosis, but only 28.7% of them associated human taeniosis with infected or undercooked pork. In the Teso North sub-county, 65.9% of respondents heard or knew about *T. solium* taeniosis, of which 55.6% associated its transmission with eating infected raw or undercooked pork. On the contrary, 57.1% of respondents from the Bunyala sub-county reported hearing about human taeniosis, but only 5% properly explained the transmission route. Only 8.2% of respondents associated *T. solium* taeniosis, human cysticercosis, and pork tapeworm, of which 19.5% of respondents from Teso North associated the diseases, while no respondent from Butula sub-county made the association between the diseases.

### WHO risk mapping

**Fig 4:**
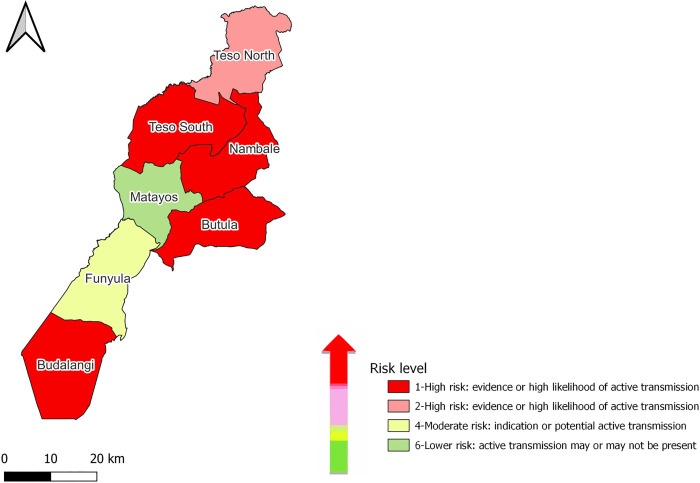
Map showing the risk of *T. solium* transmission in Busia County using the WHO mapping tool. The map was produced using the free and open-source QGIS software. The shapefile was obtained from Humanitarian Data Exchange Kenya Subnational Administrative Boundaries data set (https://data.humdata.org/dataset/geoboundaries-admin-boundaries-for-kenya) and provided by the geoBoundaries under CC BY 4.0 license https://www.geoboundaries.org/index.html#citation [42].

The WHO *T. solium* mapping tool was used for risk modeling of *T. solium* transmission in Busia County. The WHO mapping tool classified Bunyala, Teso South, Nambale, and Butula sub-counties as risk level 1 and Teso North as risk level 2 (coded by red and pink colors). Both risk levels 1 and 2 are considered high-risk areas, with a high likelihood of active transmission of *T. solium* (Fig 4). Samia sub-county was ranked as an area with moderate transmission with potential active transmission (indicated by a pale-yellow color). In comparison, the Matayos sub-county was categorized as a low-risk area where there may or may not be active transmission (highlighted by a light green color).

## Discussion

Pig rearing is a vital livelihood activity in SSA [26], and the sector showed fast growth in countries that keep pigs in the region [27]. Similarly, the pig population in Kenya has shown a linear growth since 2015. The pig distribution map identified Central and Western Kenya as regions with higher pig populations, which is comparable with the FAO report [30]. Pig production in Kenya is concentrated in areas within Central, Western, Nyanza, Eastern, Nairobi, and the Rift Valley [26,30,49]. This distribution is likely influenced by the arid and semi-arid conditions in Northern and Northeastern Kenya, as well as the Muslim-majority Coastal regions, which have made pig farming less favorable and led to a low pig population [30].

The WHO *T. solium* endemicity map [50] aligns with the TSTC distribution map generated in the current study, indicating the presence of all developmental stages of *T. solium* in Kenya. Several countries in the SSA, including Tanzania, Uganda, Rwanda, Zambia, Madagascar, Cameroon, and Burkina-Faso, have also reported the presence of TSTC [11,44,51–55].

The burden of reported cases of human cysticercosis was estimated to be 1.6% to 6.6% based on Ag-ELISA, 31.1% using Ab-ELISA, and 2.4% by EITB. Most data on human cysticercosis originates from countries outside Africa [56], and information on the disease within the continent remains limited [57]. In Zambia, the prevalence estimates range from 5.8% to 13% by Ag-ELISA and 34% to 39% by sero-antibody detection tests [58]. A relatively higher prevalence (16–45.3%) was reported from Tanzania using antigen and antibody ELISA [55].

The magnitude of *T. solium* taeniosis cases varies between 17.3% and 19.9% by microscopy and 0.18% to 6.7% using copro-antigen ELISA. Systematic reviews have reported microscopy-based prevalence rates of 0.7% in Uganda [52] and between 0.1% and 14.7% across East and Southern Africa [53]. Additionally, the prevalence range of 2.3% to 5.2% in Tanzania [55], 6.3% to 12% in Zambia, and 1.4% in Rwanda [53] was recorded based on copro-antigen ELISA. However, it is important to note that neither microscopy nor copro-antigen ELISA can distinguish between *Taenia* species [59].

Based on the included studies, the reported cases of porcine cysticercosis ranged from 1.8% to 14% by lingual examination, 3.8% to 49.9% by Ag-ELISA, and 1.8% upon meat inspection. A recent study in Kenya [40] reported 3.8% prevalence via lingual examination and 0.54% by Ag-ELISA. In Tanzania, the estimates ranged from 6% to 17.4% based on lingual examination,1.5% to 33.3% by Ag-ELISA, and 0% to 18.2% using routine meat inspection [55]. A systematic review by Gulelat *et al.* [54] estimated a pooled prevalence of 17% for porcine cysticercosis in the Eastern and Southern Africa region.

The porcine cysticercosis diagnostic tests, particularly serology, lingual examination, and meat inspection, perform poorly in estimating the true prevalence. The Ag-ELISA, while widely used, cannot distinguish between *Taenia* species and may overestimate the prevalence, with sensitivity ranging from 44.4% to 95.7% and specificity from 45% to 100% [60–62]. In contrast, lingual examination and meat inspection have high specificity (up to 100%) [61,63,64] but are poorly sensitive (22–66%) [63,64], often missing light infections and leading to underestimation. The estimates based on these methods should therefore be interpreted with caution [63,65]. Additionally, meat inspection may not reflect the true burden of porcine cysticercosis due to the widespread practice of informal pig slaughter [27,44,66].

Although several studies employed low-specific tests, the preliminary findings indicate the presence of TSTC in Kenya. The majority of studies were carried out in western Kenya, notably Busia County and parts of Siaya, Kakamega, and Bungoma counties. These areas have been the focus of TSTC investigations as they are high-risk for zoonotic diseases in the Lake Victoria crescent ecosystem [21,27,56,67] and due to free-ranging pig keeping [21,27,30,31]. In contrast, Kiambu County, with the highest pig population, recorded a lower porcine cysticercosis estimate, likely due to better sanitation and intensive pig production systems [30]. Overall, favorable environmental, socioeconomic, and husbandry factors in Western Kenya seem to support the clustering of TSTC reports, with Busia County as a key focus for understanding risk factors [15,21,40,67].

From the hospital’s retrospective data collection, no case records were found for *T. solium* taeniosis, human cysticercosis, or neurocysticercosis (NCC). The absence of NCC retrospective data may be associated with a lack of routine diagnostic facilities in rural hospitals and the limited attention given to the condition. As an alternative, retrospective epilepsy data were used as a proxy indicator for NCC, in line with the WHO *T. solium* risk mapping tool [34].

NCC is estimated to cause one-third of acquired epilepsy cases in *T. solium* endemic areas [6,68]. A study in Zambia reported a 57% NCC prevalence in people with epilepsy [12], which is higher than the global average of 30% [6] and the 22% pooled prevalence in SSA [8]. Similarly, a case-control study in western Kenya reported a higher prevalence of human cysticercosis among people with epilepsy (5%) compared to controls (2.4%) [69]. While substantial evidence supports a positive association between NCC and epilepsy in endemic areas [6–8,12,53,69,70], a study in western Kenya [56] did not find such an association. However, the finding is based on data from a single village and is not generalizable to Busia County or the wider region. Besides, given epilepsy’s diverse causes (infectious, non-infectious, and even unknown causes), the interpretation of epilepsy data as a proxy indicator of NCC should be made cautiously.

Busia County was identified as a high-risk area for TSTC through multiple layers of evidence. The map based on retrospective data revealed the spatial distribution of both porcine cysticercosis and epilepsy (as a proxy indicator for NCC) in all sub-counties. The WHO *T. solium* risk mapping tool further supported this, highlighting sub-counties such as Bunyala, Teso South, Butula, and Nambale as having a high likelihood of active transmission. The questionnaire survey revealed several risk factors, including free-range pig keeping, poor sanitary facilities, low deworming rates, limited awareness of TSTC, and so on. These are further compounded by poverty [21,31] and a low human development index [29] in the county.

Free-range pig keeping is widely practiced in Western Kenya and has been identified as a key risk factor for TSTC [14–16,21], consistent with findings from the questionnaire survey. Inadequate latrine coverage contributes to *T. solium* transmission [11,57]. Despite improvement in latrine coverage following a sanitation program [40], about one-third of respondents’ pigs had access to human excreta. Informal pig slaughter is common in Western Kenya [15,21], with 17% of respondents reporting home slaughter at least once, despite legal requirements for official inspection [21]. The survey highlighted poor community awareness of TSTC, consistent with previous reports [14,15,17,21,40,67], and this remains a major factor in *T. solium* transmission [9,11,14,15,17,21,67,71].

The WHO *T. solium* risk mapping tool identified Bunyala, Teso South, and Butula sub-counties as high-risk areas, while Matayos sub-county was classified as low-risk, consistent with the report by Chege *et al.* [40]. Similarly, mapping based on retrospective data showed that Bunyala and Butula sub-counties had the highest proportions of porcine cysticercosis and epilepsy cases, whereas Samia and Teso North had the lowest. Overall, triangulation of data from the WHO mapping tool, retrospective records, questionnaire findings, and published literature [14–16,21] confirmed ongoing transmission of TSTC in Busia County and identified sub-counties at highest risk.

Bunyala subcounty was identified as a high-risk area by the WHO *T. solium* risk mapping tool and recorded the highest proportions of cases of porcine cysticercosis and epilepsy (as a proxy indicator for NCC). The questionnaire responses in the sub-county also confirmed the presence of risk behaviors for TSTC transmission and maintenance. The majority of respondents in Bunyala sub-county practiced free-range pig production (48.6%). The sub-county had the highest report of open defecation (6%) [72], and a relatively low latrine coverage (77.1%), with most households using open-pit latrines, which are often unusable during the high rainy season. The sub-county also had the highest proportion of respondents who were unaware of TSTC transmission.

In contrast, the risk mapping tool categorized Matayos sub-county as a low-risk area where active transmission may or may not be present. Questionnaire data supported this classification by indicating relatively lower-risk behaviors. The sub-county recorded the highest proportion of confined pigs (50%) and a low rate of free-range pig keeping (7%). It also had the lowest proportion of respondents reporting pig access to sewage (17.5%) and the lowest report of open defecation (0.9%) [72]. Additionally, respondents from this sub-county demonstrated relatively higher awareness of TSTC (54%).

Generally, the presented evidence will guide targeted interventions in Busia County, and high-risk sub-counties can be prioritized for control activities, including public health education, improved latrine access, deworming campaigns, and pig confinement promotion, among others. Ultimately, the results can support national policy development and contribute to sustainable One Health approaches to interrupt the *T. solium* transmission cycle.

### Study limitation

The small number of studies from a limited geographical range and the variation in the methodological approaches were some of the limitations we encountered in compiling the literature review data. To enable comparison, only studies carried out at the community level were included for mapping of TSTC. Additionally, MSc and PhD theses were incorporated to increase the number of relevant research outputs and strengthen the dataset.

Many of the included studies employed diagnostic tests with limited accuracy, some with low specificity (e.g., Ag-ELISA, microscopy, and coproantigen ELISA) and others with low sensitivity (e.g., lingual palpation and meat inspection). These limitations may have led to overestimation or underestimation of the true estimate. Therefore, the diagnostic performance of each test needs to be considered when interpreting the results.

The lack of registered cases of NCC and *T. solium* taeniosis from the county and sub-county hospitals posed a challenge in the risk mapping of TSTC in Busia County. As an alternative, retrospective epilepsy data were used as a proxy indicator of NCC.The lack of accurate data on the total number of patients examined for epilepsy cases was also a challenge in estimating the proportion of epilepsy. Due to this limitation, the number of patients who visited the hospitals within the same duration of epilepsy cases was used as the denominator.

The data used for mapping TSTC distribution at the national level (*i.e.,* literature review) has an over-decade-long life span. Whereas the data obtained through retrospective and prospective approaches for mapping the distribution of TSTC in Busia County were collected in relatively comparable time spans. Hence, the interpretation should consider these differences. It should be noted that the collected pig population data did not distinguish between different pig production systems, limiting its scope to the provision of background information.

Initially, both Busia and Kiambu County were chosen as the study areas due to their high pig population. However, movement restrictions were imposed due to an outbreak of foot-and-mouth disease in all sub-counties of Kiambu County and later by COVID-19 at the national level. As a result, we were unable to return to these areas to complete data collection.

## Conclusion and recommendation

Most reported TSTC cases were from Kenya’s Western region, which has a relatively higher porcine cysticercosis report. Different mapping approaches pointed out the high likelihood of active TSTC transmission in Bunyala, Teso South, Butula, and Nambale sub-counties. The WHO *T. solium* risk mapping tool categorized the Matayos sub-county as a low-risk area. The questionnaire survey also supported the spatial maps’ findings and highlighted the status of key risk factors in the county. Generally, the study indicated the presence of TSTC transmission in Western Kenya, particularly in Busia County, where effective control strategies are required. Though data based on definitive diagnoses should be collected to confirm TSTC endemicity, the result identifies high-risk areas where control intervention should be implemented to control TSTC. Local authorities and concerned stakeholders can use these findings to inform strategies aimed at improving public awareness about TSTC, deworming campaigns, promoting safe slaughter practices, pig confinement promotion, etc. Additionally, the study employed a mixed-methods approach, integrating routinely collected data with open-source resources. By compiling and analyzing these data, new evidence was generated. This approach can be applied in other resource-limited settings and has the potential to contribute to sustainable One Health strategies aimed at interrupting the *T. solium* transmission cycle.

## Supporting information

S1 Table Kenya’s pig population between 2015–2020 based on FAOSTAT data. (PDF)

S2 Table Sociodemographic characteristics of the respondents. (PDF)

S1 Data ODK questionnaire survey data. (XLSX)

S2 Data WHO *T. solium* risk classification template used for Busia County. (PDF)

S3 Data Pig population distribution data collected from key pig-keeping counties. (XLSX)
