# Correction: The epidemiology and spatial distribution of *Taenia solium* taeniosis and cysticercosis in Kenya: The case of Busia County

**DOI:** 10.1371/journal.pntd.0013957

**Published:** 2026-02-02

**Authors:** Yewubdar Gulelat Zemedhun, Tadesse Eguale, Nigatu Kebede Wubie, James M. Akoko, Eric M. Fèvre, Elizabeth A. J. Cook

The second author’s name is spelled incorrectly. The correct name is: Tadesse Eguale. The correct citation is: Zemedhun YG, Eguale T, Wubie NK, Akoko JM, Fèvre EM, Cook EAJ (2025) The epidemiology and spatial distribution of *Taenia solium* taeniosis and cysticercosis in Kenya: The case of Busia County. PLoS Negl Trop Dis 19(12): e0013746. https://doi.org/10.1371/journal.pntd.0013746.
