# Supplementary material for: The epidemiology and spatial distribution of Taenia solium taeniosis and cysticercosis in Kenya: The case of Busia County
Source: PLoS Negl Trop Dis. 2025 Dec 5;19(12):e0013746. doi: 10.1371/journal.pntd.0013746 (PMC12680180; doi:10.1371/journal.pntd.0013746)
Supplement: S1 Table — (PDF) [file pntd.0013746.s001.pdf]

Kenya pig population data between 2015-2020 based on FAOSTAT

Year	Total no of pigs			
2015	462033			
2016	504395			
2017	554301			
2018	567843			
2019	596414			
2020	649273			
