# Supplementary material for: The epidemiology and spatial distribution of Taenia solium taeniosis and cysticercosis in Kenya: The case of Busia County
Source: PLoS Negl Trop Dis. 2025 Dec 5;19(12):e0013746. doi: 10.1371/journal.pntd.0013746 (PMC12680180; doi:10.1371/journal.pntd.0013746)
Supplement: S2 Table — (PDF) [file pntd.0013746.s002.pdf]

Demographic characteristics of respondents from Busia County, Kenya

Characteristics	Category	Frequency	Percent (%)
Sex	Female	107	37.7
	Male	177	62.3
Respondent age-group	≤24	23	8.1
	25–34	66	23.2
	35–44	92	32.4
	45–54	50	17.6
	55–64	26	9.2
	>64	27	9.5
Educational level	None	41	14.4
	No formal education but can read and write	6	2.1
	Primary	153	53.9
	Secondary	63	22.2
	Tertiary	21	7.4
