# Supplementary material for: The epidemiology and spatial distribution of Taenia solium taeniosis and cysticercosis in Kenya: The case of Busia County
Source: PLoS Negl Trop Dis. 2025 Dec 5;19(12):e0013746. doi: 10.1371/journal.pntd.0013746 (PMC12680180; doi:10.1371/journal.pntd.0013746)
Supplement: S2 Data — (PDF) [file pntd.0013746.s004.pdf]

S2 Data. WHO *T. solium* risk classification template used for Busia County

Country data

**COUNTRY**	**Kenya**
Year	**2023**
How many administrative units (at the lower level) you want to map?	7
Name of person filling the mapping tool	Yewubdar Gulelat
Date report	16-Aug

Taenia solium active transmission risk area mapping: 2023

COUNTRY/REGION Kenya **Filled by:** Yewubdar Gulelat **Date:** 16/08/2023

Name of administrative level 1 (State, Province, Region)	Name of administrative level 2 (District, Municipality, Village)	**Disease**				**Risk factors**		Risk level	Comments
		Porcine cysticercosis	Taeniasis by *T. solium*	Taeniasis	Neuro-cysticercosis	Open defecation	Backyard pigs common		
Busia County	Nambale	Yes	Unknown	Unknown	Yes	Unknown	Yes	**1**	
Busia County	Butula	Yes	Unknown	Unknown	Yes	Unknown	Yes	**1**	
Busia County	Bunyala	Yes	Unknown	Unknown	Yes	Yes	Yes	**1**	
Busia County	Teso-South	Yes	Unknown	Yes	Yes	Unknown	Yes	**1**	
Busia County	Teso-North	Unknown	Unknown	Yes	Yes	Unknown	Yes	**2**	
Busia County	Samia	Unknown	Unknown	Unknown	Yes	Yes	Yes	**4**	
Busia County	Matayos	Yes	Unknown	Unknown	Yes	Unknown	No	**6**	

Risk classification levels for areas suspected endemic to *Taenia solium*

CRITERIA TABLE

Yes - Present; **Ø** - Might be present; **?** - Not confirmed or unknown

Updated 31/03/2021

Disease				Risk factors		Risk level	Comments
Porcine cysticercosis	Taeniasis by *T. solium*	Taeniasis	Neuro-cysticercosis	Open defecation	Backyard pigs common		
Yes	Ø	Ø	Ø	?	?	**1**	If pigs autochthonous, indicates active transmission present
	Yes	Ø	Ø	?	Yes	**1**	
	Yes	Ø	Ø	Yes	?	**1**	Presence of backyard pigs not confirmed (but likely)
	Yes	Ø	Yes	?	?	**2**	
		Yes	Ø	Yes	Yes	**2**	
		Yes	Ø	?	Yes	**2**	Maybe still open defecation in fields or latrines not used
		Yes	Yes	?	?	**3**	
			Yes	Yes	Yes	**3**	
		Yes		Yes	?	**4**	Could be *T. saginata*
				Yes	Yes	**4**	*T. solium* might not be necessarly present
			Yes	?	Yes	**5**	
			Yes	Yes	?	**5**	
	Yes	Ø	?	?	?	**5**	Taeniasis could have been acquired somewhere else
		Yes	?	?	?	**6**	Could be *T. saginata*
Yes	Ø	Ø	Ø	?	No	**6**	Presumed porcine cysticercosis in imported pigs or pork
				Yes	?	**NS**	
				?	Yes	**NS**	

NOTE: Not every permutation is either likely or represented

Risk color legend:

1 - 2	High risk: **evidence** or high likelihood of active transmission
3 - 4	Moderate risk: **indication** or potential active transmission
5 - 6	Lower risk: active transmission **may or may not** be present
NS	Not sufficient information to classify risk
